# Immunomodulatory effects of gut microbiota on vaccine efficacy against respiratory pathogens

**DOI:** 10.3389/fimmu.2025.1618921

**Published:** 2025-06-03

**Authors:** Li Xue, Chunhua Wang, Chuanyu Liu

**Affiliations:** ^1^ Department of Scientific Research, Xiangyang Central Hospital, Affiliated Hospital of Hubei University of Arts and Science, Xiangyang, China; ^2^ Department of Clinical Laboratory, Xiangyang No.1 People’s Hospital, Hubei University of Medicine, Xiangyang, China; ^3^ Central Laboratory, Xiangyang Central Hospital, Affiliated Hospital of Hubei University of Arts and Science, Xiangyang, China

**Keywords:** gut microbiota, respiratory pathogens, vaccine efficacy, gut-lung axis, immunomodulatory effects

## Abstract

The outbreaks of respiratory pathogens like severe acute respiratory syndrome coronavirus 2 (SARS-CoV-2) and influenza virus (IV) have heightened the demand for highly effective vaccines that provide strong and durable immunity in human populations. However, immune responses to vaccination vary significantly among individuals and populations. Recent studies have demonstrated that the gut microbiota play an essential role in regulating respiratory pathogens vaccination-induced immune responses through the systemic effects of gut-lung axis on distant organs, the lungs. In this review, we first synthesize the changes in gut microbiota composition and immune responses that occur during respiratory pathogen infections and vaccination. Then, we discuss the underlying immunological mechanisms of bidirectional immunomodulatory effects between gut microbiota and vaccines. Finally, we explore the strategies for designing next-generation vaccines against respiratory pathogens in term of gut microbiota-mediated immunological pathway.

## 1 Introduction

Vaccines are widely acknowledged as one of the most effective interventions for the prevention of respiratory infectious diseases, including coronavirus disease 2019 (COVID-19). Clinical evidence substantiates that COVID-19 vaccines can mitigate disease severity upon infection with severe acute respiratory syndrome coronavirus 2 (SARS-CoV-2) (1). It is well-established that vaccines primarily confer protection by inducing B cells to produce neutralizing antibodies and by stimulating T cells to recognize and eliminate infected cells (2). However, the immune responses elicited by vaccination exhibit considerable variability among individuals and populations. For example, the antibody titers induced by the trivalent influenza vaccine (TIV) and the hepatitis B vaccine can differ by as much as 100-fold between individuals (3, 4). This variability influences both the protective efficacy of the vaccine and the duration of the conferred protection. Consequently, identifying the underlying causes of this variability is essential for enhancing vaccine effectiveness.

Recent animal experiments and clinical studies have demonstrated that gut microbiota play a crucial role in the development and regulation of immune responses, thereby affecting vaccine efficacy (4, 5). Specifically, there exists a bidirectional relationship between gut microbiota and the COVID-19 vaccine, wherein variations in microbiota composition can either enhance or diminish vaccine-induced efficacy, while COVID-19 vaccines may reduce the overall number and diversity of microbial organisms (6, 7). However, the implications of this bidirectional relationship for vaccine immunity and safety remain inadequately understood. Elucidating the fundamental mechanisms by which gut microbiota influence vaccine responsiveness is essential for optimizing respiratory vaccines.

The gut microbiota comprises trillions of complex commensal microorganisms, which not only involve in digestion and nutrient metabolism but also influence immune homeostasis and pathogen defense (8). This concept is illustrated in murine models, where antibiotic-induced disruption of the gut microbiome balance prior to respiratory infection leads to increased inflammation and mortality during the infection (9–12). Recent evidence increasingly supports the association between the gut microbiota and respiratory infections through critical crosstalk between the gut and lungs, commonly referred to as the ‘gut-lung axis’ (13–15). For instance, the immune responses of individuals infected with respiratory pathogens are linked to the bidirectional interactions between the gut microbiota and lungs (9). Notably, during SARS-CoV-2 infection, the gut microbiota and intestinal barrier function are compromised, potentially allowing bacterial components and toxins to enter the bloodstream and exacerbate systemic inflammation. Furthermore, dysbiosis of the gut microbiota may impair the recruitment of immune cells to the lungs, thereby increasing proneness to respiratory infections. Consequently, interventions targeting the microbiome, including vaccines and antibodies, may present innovative therapeutic and preventive strategies for infections caused by respiratory pathogens.

In this review, we initially synthesize the latest evidence from both animal models and clinical cohort studies regarding the bidirectional immunomodulatory interactions between gut microbiota and respiratory pathogens vaccine. Subsequently, we examine the underlying immunological mechanisms that mediate these interactions and influence the immunogenic efficacy of the vaccines. Finally, we investigate design strategies for next-generation vaccines against respiratory pathogens, focusing on the immunological pathways mediated by gut microbiota.

## 2 The changes in gut microbiota composition and immune responses during respiratory pathogen infections

The gut and respiratory tract are recognized as two primary mucosal immune systems, sharing a common embryonic origin and exhibiting similar structural characteristics. Together, their functions are complementary, working in concert to maintain the body’s normal physiology (16, 17). Individuals who contract infections from respiratory pathogens at different intervals, such as SARS-CoV-2 and influenza virus (IV), exhibit altered intestinal function and structure, primarily manifesting as intestinal microbiome dysbiosis, increased intestinal permeability, and enterocyte damage. Furthermore, these disruptions may exacerbate the progression and outcomes of respiratory diseases, suggesting the existence of complex bidirectional interactions between the gut and lung (9, 18, 19). Although current studies have implicated the lymphatic system as a critical pathway of the gut-lung axis, through which the gut microbiota modulates respiratory disease progression and host immune responses, the precise underlying mechanisms of these interactions remain to be elucidated (20). To address this gap, an increasing number of researchers have concentrated on investigating the interactions between gut microbiota and respiratory viral infections, particularly concerning the COVID-19 and IV, which have posed persistent threats to global health in recent years.

### 2.1 SARS-CoV-2 infection

Alterations in the gut microbiome, marked by changes in microbial diversity and abundance, can substantially affect the body’s ability to combat viral infections. For example, antibiotic-induced dysbiosis in gut microbiota significantly compromises both innate and adaptive immune responses during respiratory viral infections (11). An increasing number of studies have also observed that COVID-19 patients exhibit reduced bacterial diversity, an increased presence of opportunistic pathogens, and a decreased abundance of beneficial symbionts in their fecal microbiota compared to healthy individuals (21–23).

In individuals afflicted with COVID-19, alterations occur in both bacterial and viral populations, with distinct microbial compositions correlating with different levels of disease severity. Utilizing a murine model of COVID-19, further research indicates that SARS-CoV-2 infection induces differential expression of genes related to immunity and infection within gut epithelial cells (23). Moreover, pre-existing abnormalities in gut microbiota may lead to the downregulation of angiotensin-converting enzyme 2 (ACE2) expression, thereby influencing proneness to SARS-CoV-2 infection (13).

### 2.2 Influenza A virus infection

IAV infection similarly disrupts the homeostasis of the gut microbiota. Murine studies have demonstrated that levels of *Escherichia coli*, *Helicobacter hepaticus*, and *Clostridium perfringens* are elevated, while levels of *Desulfovibrio C21_c20* and *Lactobacillus salivarius* are reduced (24). Further in-depth research has suggested that this dysbiosis is not merely a consequence of IAV infection but plays a crucial role in influencing subsequent antiviral immunity. Existing studies have shown that, in addition to affecting the translocation of intestinal innate lymphoid cells to the pulmonary system, the gut microbiota activates innate immunity through Toll-like receptors (TLRs), thereby enhancing antiviral defenses. Collectively, these processes establish a gut-lung axis that influences IAV pathogenesis, disease severity, and outcomes by modulating immune balance and maintaining epithelial integrity (14). In recent years, a growing body of intervention studies has concentrated on the interactions between gut microbiota and respiratory pathogen infections. For example, the gut microbiota known as *segmented filamentous bacteria* has been shown to decrease viral titers by modulating resident alveolar macrophages (AMs) and enhancing the expression of C1qa, thereby conferring protection against influenza infection in murine models (15).

In summary, these findings presented herein substantially enhance our comprehension of the infection processes of respiratory pathogens and the alterations of immune homeostasis mediated by gut microbiota on the distal organ, the lung. Furthermore, these studies have the potential to contribute to the development of innovative therapeutic strategies, such as the modulation of gut microbiota to bolster host defense mechanisms and improve outcomes in viral respiratory infections.

It is essential to recognize that respiratory vaccines remain the most consistently effective measure for preventing viral infections. By elucidating and leveraging the complex interactions between the immune system and microbiota, it may be possible to develop more effective vaccines tailored to individual microbiota profiles, ultimately leading to improved health outcomes.

## 3 Bidirectional immunomodulatory effects between gut microbiota and respiratory pathogens vaccine

The composition of gut microbiota varies significantly among individuals, leading to considerable variability in the immunogenicity of respiratory pathogens vaccines (1, 25). In the following sections, we examine evidence demonstrating that modifications in gut microbiota substantially influence immune responses to a range of respiratory vaccines, as observed in both preclinical animal studies and human clinical trials (see **Table 1**).

**Table 1:** Summary of the effect of gut microbiota on the respiratory pathogen vaccine.

Study type	Animals/Locations	Vaccine	Results related to vaccine efficacy	References
Animal study	Mice	BCG vaccine	Gut dysbiosis notably reduced the immune response to BCG and hindered the clearance of *Mycobacterium tuberculosis (Mtb).*	(26)
Animal study	Mice	Pneumococcal vaccine	The gut IgA is positively associated with the pneumococcal vaccine.	(27)
Prospective cohort study	Hong Kong	CoronaVac and BNT162b2 vaccine	*Bifidobacterium adolescentis* was positively related to the neutralizing antibodies of the CoronaVac vaccine; the overall abundance of bacteria with flagella and fimbriae, like *Roseburia faecis*, was positively correlated with neutralizing antibodies to the BNT162b2 vaccine; an increased abundance of *Prevotella copri* and two *Megamonas* species were reported in subjects experienced fewer adverse incidents with the aforementioned two vaccines.	(6)
Prospective cohort study	Hong Kong	Three doses of the CoronaVac vaccine	*Eubacterium rectale*, *Collinsella aerofaciens*, and Streptococcus salivarius were inversely related to the sustained immune response following the administration of three doses of CoronaVac.	(28)
Prospective cohort study	USA	SARS-CoV-2 mRNA Vaccine	The phylum *Desulfobacterota* and genus *Bilophila* showed a positive correlation with IgG, whereas *Bacteroides* exhibited a negative correlation.	(29)
Prospective cohort study	The Republic of Korea	two doses of BNT162b2/ChAdOx1and one dose of BNT162b2 vaccine	*Faecalibacterium prausnitzii* was linked to the strong and lasting antibody responses following BNT162b2 vaccination, whereas *Escherichia coli* was linked to a slower antibody decline in post-ChAdOx1 vaccination.	(30)
Prospective cohort study	The Republic of Korea	BNT162b2/ChAdOx1 vaccine	The initial abundance of the genus *Parasutterella* and the species *Eubacterium PAC001034_s* and *Blautia_uc* positively correlated with humoral immune responses to the ChAdOx1 vaccine; the levels of the genera *Ruminococcaceae PAC000661_g*, *Romboutsia*, and *Lachnospiraceae PAC001043_g* and species *Clostridium PAC001136_s*, *Lachnospiraceae PAC001043_g PAC001449_s*, *Eubacterium LT907848_s*, *Romboutsia timonensis*, and *Roseburia cecicola* showed a positive correlation with immune response to the BNT162b2 vaccine; the *Lachnospiraceae* family may be positively linked to an elevated immune response in individuals receiving both above vaccine.	(31)
Prospective cohort study	The Netherlands	BCG vaccine	*Roseburia* exhibits a negative correlation with BCG-induced nonspecific trained immunity, whereas the specific T cell-mediated memory responses show positive associations with *Eggerthella lenta* and *Ruminococcus*. The identified immunomodulatory taxa exhibit significant influence on circulating metabolites, with *Roseburia* notably impacting phenylalanine metabolism.	(32)
Prospective cohort study	GA and Atlanta	Trivalent influenza vaccine	While individuals with pre-existing high antibody titers showed no significant alteration in antibody responses, those with low baseline titers ​displayed compromised H1N1-specific neutralization capacity alongside diminished IgG1 and IgA binding activity.	(33)
Prospective cohort study	China	Influenza A	Nine microbial taxa, such as genus *Bilophila*, species *Bifidobacterium longum*, genus *Collinsella*, genus *Ruminococcus*, family *Veillonellaceae*, species *Bifidobacterium longum*, phylum *Bacteroidetes*, class *Bacteroidia*, and order *Bacteroidales*, demonstrated a positive correlation with serum anti-IAV IgG levels, while the other three taxa, including genus *Lachnospiraceae noname*, genus *Coprococcus*, and species *Lachnospiraceae bacterium 31_46FAA* were negatively associated.	(34)

### 3.1 Evidence from animal models

Recent research has underscored the pivotal role of gut microbiota as a mediator in the regulation of the immune system, significantly affecting vaccine efficacy through its modulatory effects on systemic immune responses. For instance, one study examined the impact of gut dysbiosis on the efficacy of the Bacillus Calmette-Guérin (BCG) vaccine, revealing that disturbances in gut microbiota can attenuate immune responses. In *Mtb*-infected mice, this dysbiosis resulted in impaired production of effector and memory T cells, leading to an increase in colony-forming units in the lungs (26). Similarly, another study indicated that early-life gut disruption caused by antibiotic treatment in infant mice significantly impaired the antigen-specific IgG response. However, this impaired antibody response was restored following the reestablishment of the commensal microbiota. Interestingly, this impairment was not observed in adult mice subjected to the same antibiotic treatment (35).

In addition to the impaired immune responses observed in specific pathogen-free (SPF) mice treated with antibiotics, vaccination-induced immune responses were also correlated with mice exposed to diverse microbial environments (referred to as “dirty mice”) (36, 37). Fiege et al. conducted an assessment of the differential immune responses between SPF and dirty mice following immunization with influenza vaccines (37). Their findings indicated that dirty mice more accurately model human responses to vaccination, as evidenced by the similar human transcriptional signatures observed in these mice. Furthermore, in comparison to SPF mice, the humoral and cellular responses induced by influenza vaccination in dirty mice were attenuated, resulting in decreased protective efficacy.

Parenteral BCG vaccination exerts a notable influence on the gut microbiota and tissue-resident memory macrophages in the lungs. In an experimental study, Jeyanathan et al. explored the mechanisms underlying immune responses in the lung following subcutaneous BCG vaccination. Their findings revealed that the gut microbiota, metabolome, and barrier function underwent time-dependent alterations due to *mycobacterial* dissemination (38). Subsequently, changes in the serum and lung metabolome were observed via the gut-lung axis of distal mucosal pathway, leading to the induction of memory AMs and trained immunity in the lung. Another study reported that subcutaneous BCG vaccination modified both the alpha- and beta-diversity of the gut and lung microbiomes (39). Notably, the diversity of the lung microbiota was most significantly affected, potentially contributing to enhanced defense mechanisms against tuberculosis infection. Furthermore, the role of gut microbiota in vaccine-induced immune responses is not confined to BCG vaccines but extends to other vaccines as well. Recent findings indicate that gut-derived IgA, produced in response to intestinal B cell interactions with commensal bacteria, enhances systemic IgG responses to pneumococcal vaccines (27). These results elucidate a mechanistic framework for utilizing microbial communities to modulate vaccine immunogenicity and offer a novel perspective on the development of innate immune memory in the lungs through pathways mediated by gut microbiota.

### 3.2 Correlative evidence from clinical cohort studies

The relationship between gut microbiota and the efficacy of various vaccines has emerged as a topic of increasing interest, especially within the framework of clinical cohort studies.

#### 3.2.1 COVID-19 vaccines

To mitigate the COVID-19 pandemic, a range of vaccines were developed and authorized, including mRNA vaccines, protein subunit vaccines, non-replicating viral vector vaccines, and inactivated virus vaccines, all designed to elicit effective immune responses to protect against COVID-19 infection (2). Numerous studies have indicated that the gut microbiota significantly influences the efficacy and safety of COVID-19 vaccines (6, 28–31, 40, 41). Conversely, COVID-19 vaccines have also been shown to impact the composition and abundance of gut microbiota (2).

A recent prospective cohort study identified a positive correlation between baseline microbial species richness and humoral immunogenicity in adults who received full vaccination with the BNT162b2 and ChAdOx1 (adenovirus-vectored) vaccines. The *Lachnospiraceae* family was potentially associated with an enhanced immune response in individuals vaccinated with both the ChAdOx1 and BNT162b2 vaccines (31). Another prospective cohort study, which aimed to investigate the relationship between gut microbiota and immunogenicity following the administration of three doses of CoronaVac in Hong Kong, found that *Eubacterium rectale*, *Collinsella aerofaciens*, and *Streptococcus salivarius* were inversely related to the sustained immune response (28). Consistent with these findings, microbial diversity was associated with final IgG levels. However, only the pre-vaccination microbial composition and anticipated function were correlated with the vaccine’s effectiveness. Specifically, the phylum *Desulfobacterota* and the genus *Bilophila* demonstrated a positive correlation with IgG levels, while *Bacteroides* exhibited a negative correlation (29).

Furthermore, the gut microbiome has been implicated in the safety and efficacy of vaccines. A recent prospective observational study investigated the relationship between gut microbiota composition and both the immunogenicity and adverse events associated with COVID-19 vaccines. The study revealed that recipients of the CoronaVac vaccine demonstrated a significantly weaker immune response compared to those who received the BNT162b2 vaccine. Notably, a higher abundance of *Bifidobacterium adolescentis* was observed in individuals with elevated levels of neutralizing antibodies following CoronaVac vaccination. Conversely, the presence of bacteria with flagella and fimbriae, such as *Roseburia faecis*, was positively associated with neutralizing antibody levels in individuals vaccinated with BNT162b2. Additionally, an increased abundance of *Prevotella copri* and two *Megamonas* species was identified in individuals experiencing fewer adverse events with both vaccines (6). It is important to note that the level of neutralizing antibodies tends to decline over time post-vaccination. The gut microbiome may play a crucial role in modulating the longevity of vaccine-induced antibody responses. A prospective cohort study conducted in the Republic of Korea has demonstrated that *Faecalibacterium prausnitzii* is associated with robust and sustained antibody responses following BNT162b2 vaccination, while *Escherichia coli* is associated with a slower decline in antibody levels following ChAdOx1 vaccination (30).

A study conducted within a large population-based cohort intriguingly identified specific microbial signatures associated with variations in antibody levels following vaccination (42). These findings discussed above have identified the specific gut microbiota biomarkers associated with vaccine outcomes, potentially offering critical insights for the advancement of effective personalized vaccination strategies. Nevertheless, these specific biomarkers differ across various vaccine types. Therefore, further research is necessary to determine how targeted modulation of these microbial markers could optimize vaccine efficacy while minimizing adverse reactions.

Overall, these studies demonstrate that gut microbiota play a crucial role in influencing the efficacy and safety of COVID-19 vaccines. A comprehensive understanding of these associations may facilitate the development of improved vaccination strategies and personalized approaches, thereby potentially enhancing immune responses and vaccine effectiveness across diverse populations.

#### 3.2.2 BCG vaccine

Accumulating evidences suggest that gut microbial communities serve as key mediators of immunological processes, particularly in modulating vaccine responsiveness and maintaining immune competence. A metagenomic study involving 321 healthy adults revealed that variations in microbial genomic abundance are associated with heterogeneity in cytokine production following BCG vaccination, which in turn affects circulating metabolites. Notably, the genus *Roseburia* demonstrates a negative correlation with BCG-induced nonspecific trained immunity, whereas specific T cell-mediated memory responses show positive associations with *Eggerthella lenta* and *Ruminococcus* (32). Several studies have demonstrated that BCG vaccination can enhance microbial responsiveness while reducing systemic inflammation (43). These findings suggest that the complex interplay between gut microbiota and the immune system can influence the body’s response to vaccinations by modulating systemic inflammation and immune memory.

#### 3.2.3 Influenza vaccine

The current understanding of the interplay between the gut microbiome and vaccines is predominantly based on murine models, with limited translational evidence available for human populations. To address this gap in knowledge, Hagan et al. conducted a longitudinal study (33). In this study, broad-spectrum antibiotics were administered to healthy adults both before and after influenza vaccination, resulting in a significant reduction in gut bacterial load. Participants with high pre-existing antibody titers exhibited no significant changes in antibody responses; however, those with low baseline titers demonstrated a compromised H1N1-specific neutralization capacity, along with reduced IgG1 and IgA binding activity. These findings indicate that the gut microbiota plays a crucial role in modulating the immune response to influenza vaccines, particularly in individuals with low baseline immunity (44). Furthermore, using two-sample bidirectional Mendelian randomization, the study identified 12 microbial taxa and 14 functional pathways that are causally associated with anti-IAV IgG levels following vaccination. In a noteworthy finding, nine taxa, including the genus *Bilophila*, species *Bifidobacterium longum*, genus *Collinsella*, genus *Ruminococcus*, family *Veillonellaceae*, species *Bifidobacterium longum*, phylum *Bacteroidetes*, class *Bacteroidia*, and order *Bacteroidales*, exhibited a positive correlation with serum anti-IAV IgG levels. Conversely, three other taxa, namely genus *Lachnospiraceae noname*, genus *Coprococcus*, and species *Lachnospiraceae bacterium 31_46FAA*, were negatively associated (34). These analyses provide novel insights into the causal relationship between gut microbiota and immunogenicity. Post-vaccination, modulating gut microbiota may offer a promising strategy for enhancing human immunity and thereby preventing viral infections.

Moreover, evidence indicating that gut microbiota modulate vaccine immunogenicity is further supported by interventions involving prebiotics and probiotics. A systematic review summarizing the effects of probiotics on responses to the TIV identified that six probiotics enhanced immunogenicity, one probiotic partially improved immunogenicity, and three probiotics did not enhance immunogenicity (45). In terms of prebiotic interventions, a study demonstrated that the intervention group, which received a formula enriched with two specific prebiotics and fermented milk products, showed a potential prolongation in the duration of antibody titers against the H1N1 strain. This effect was attributed to the promotion of intestinal *Bifidobacterium* populations, as compared to the control group (46). Additionally, an observational study examined the impact of dietary fiber (DF) intake on the humoral immune response to the TIV, finding a positive correlation between improved vaccine responses and the proliferation of fiber-fermenting microbiota in the gut (47). However, this correlation did not reach statistical significance and was observed only in individuals receiving the vaccine for the first time.

## 4 Potential mechanisms of the bidirectional immunomodulatory effects

**Figure 1:**
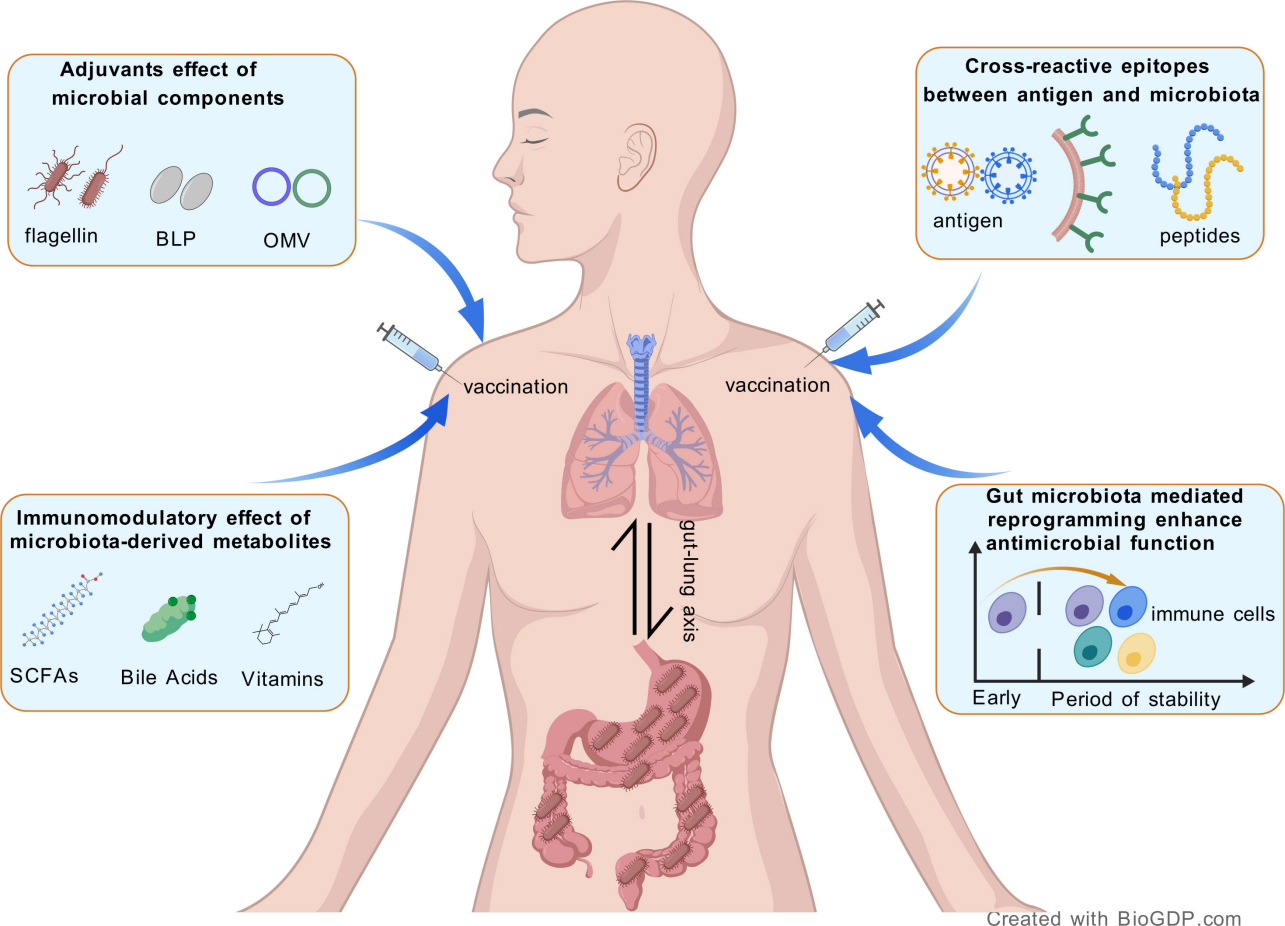
Schematic representation of the potential mechanisms between the gut microbiota and respiratory vaccines.

The mechanisms underlying the bidirectional immunomodulatory interactions between gut microbiota and respiratory pathogen vaccines remain incompletely elucidated. Recent research has underscored that the components and metabolites of gut microbiota have the capacity to modulate vaccine immunogenicity by affecting both innate and adaptive immune responses. In this context, we present potential mechanistic studies exploring the influence of gut microbiota on the immune efficacy of vaccines (**Figure 1**).

### 4.1 Natural adjuvants effect of microbial components

To enhance antigen-specific immune responses, adjuvants, which are additional immunostimulatory agents, were incorporated into non-live vaccines, including inactivated vaccines, protein subunit vaccines, and virus-like particle vaccines (48). Certain natural extract components produced by the gut microbiota, such as lipopolysaccharide (LPS), flagellin, and peptidoglycan, function as vaccine adjuvants by activating pattern recognition receptor (PRR) pathways in antigen-presenting cells (49). For instance, the level of antibody responses to non-adjuvanted vaccines (e.g., TIV) has been shown to correlate with the early expression levels of TLR5 stimulated by gut microbiota-derived flagellin (50). This phenomenon may be attributed to the differentiation of plasma cells promoted by TLR5-mediated sensing of flagellin. Furthermore, to improve the immunogenicity and efficacy of recombinant influenza vaccines, certain flagellin-based vaccines have been developed. These vaccines are designed to exert adjuvant effects by immunizing a fused protein that includes hemagglutinin (HA) and the TLR5 ligand flagellin (51, 52). In recent years, bacterial-like particles (BLPs) derived from *lactic acid bacteria* have been utilized as vaccine antigen delivery carriers against infectious diseases, such as the IV (53, 54). Composed of a peptidoglycan outer layer, BLPs have been identified as a novel adjuvant capable of activating the innate immune system, primarily through TLR2, in the context of nasal vaccines (55). Intranasal administration of BLPs combined with an antigen has been shown to elicit a robust mucosal IgA response in mice, providing complete protection against both homologous and heterologous IV challenges (56). The alternative TLR4 agonist, monophosphoryl lipid A (MPLA), derived from the LPS of the microbiota *Salmonella Minnesota*, has been employed as an adjuvant in the licensed human papillomavirus (HPV) vaccine Cervarix^®^ (57, 58). Additionally, outer membrane vesicles (OMVs), which are released by gram-negative bacteria, have emerged as a promising vaccine platform for combating various pathogens (59). Several OMV-based vaccines are currently licensed and undergoing clinical trials, including those targeting COVID-19 (60) and *Haemophilus infuenzae* type b (61). These vaccines, which mimic the bacterial surface and contain multiple surface-exposed antigens, effectively stimulate the adaptive immune response by exerting a self-adjuvant effect (62). Nonetheless, OMV-based vaccines face several challenges, such as issues related to production and purification in large-scale applications (63), as well as the low expression levels of heterologous proteins on the OMV surface (59).

### 4.2 Immunomodulatory effects of microbiota-derived metabolites

In addition to the microbial components, metabolites derived from the microbiota can also enter the systemic circulation, thereby influencing and modulating immunological responses (64). Short-chain fatty acids (SCFAs), which are produced by gut microbiota as fermentation products of DF, function as commensal-derived stimulators that regulate host antibody responses. In mice with low SCFA production, antigen-specific antibody responses were found to be impaired but could be restored through the intake of SCFAs or DF (65). Mechanistically, SCFAs regulate gene expression and enhance cellular metabolism and plasma B cell differentiation, thereby facilitating host antibody responses to vaccines. In vancomycin-treated mice immunized with the rabies vaccine, antigen-specific virus-neutralizing antibody levels were elevated through oral administration of butyrate-producing bacteria and exogenous butyrate supplementation (66). Furthermore, butyrate supplementation promoted and augmented the generation of germinal center (GC) B cells, plasma cells (PCs), and antigen-specific antibody-secreting cells (ASCs). A study was also reported that the immune response to inactivated COVID-19 vaccine positively correlated with the production microbiome-related SCFA (67). Specifically, participants with a high antibody response to the BBIBP-CorV vaccine exhibited significantly higher levels of SCFAs compared to those with a low response. In addition to SCFAs, other microbiota-derived metabolites, such as bile acids, have been shown to regulate immune responses (68). For example, an extensive study examined the innate and adaptive immune responses of healthy adults to the trivalent inactivated influenza vaccine following antibiotic-induced depletion of the gut microbiota (33). The findings revealed a significant decrease in antibody responses among subjects with low pre-existing immunity to influenza, which was accompanied by enhanced inflammatory signatures and reduced levels of bile acids. Conversely, a recent study reported that the high hepatitis B vaccine responses in the biliary atresia (BA) children has shown to significantly decreased levels of bile acids (69). Furthermore, serum bile acid concentrations demonstrate an inverse correlation with post-class-switched memory B cells. Given the inconsistent data, further research is necessary to elucidate whether bile acids augment or inhibit vaccine-induced humoral immunity.

### 4.3 Cross-reactive epitopes between antigen and microbiota

A previous study found that the proportion of CD4^+^ memory T cells specific to viral antigens in peripheral blood was abundant, even in adults who had never been infected with the IV (70). One potential mechanism is that influenza-reactive T lymphocytes can cross-recognize gut microbial peptides. In addition to gut microbiota, the oral microbiota can also modulate antibody responses against the SARS-CoV-2 Spike protein (71). Specifically, the oral bacterium *Streptococcus salivarius* can induce neutralizing monoclonal antibodies by expressing RSSL-01370 proteins that mimic the viral receptor-binding domain (RBD), thereby facilitating SARS-CoV-2 clearance. In vaccinated individuals, the IgG antibodies in saliva were enhanced by an increased abundance of *Streptococcus salivarius*. Another study demonstrated that commensal gut bacteria can cross-react with the SARS-CoV-2 S2 antibody, thereby influencing the immune response to vaccines (72). Taken together, these data suggest that cross-reactive epitopes exist in both antigens and microbiota, providing potential evidence to support or impair vaccine responses.

### 4.4 Gut microbiota mediated reprogramming to enhance the immunogenicity of vaccine

The gut microbiota plays a crucial role in the developmental programming of the immune system. Early-life microbial exposure can significantly impact long-term immune function (73). During this critical developmental period, the dynamic interaction between the microbiota and the immune system mutually influences their maturation: colonization by commensal microbes facilitates the formation of lymphoid tissues and the functional maturation of lymphocytes, while the host’s immune system actively shapes the composition and stability of the microbial community, maintaining a harmonious balance within the ecosystem (74). For instance, microbial exposure during the neonatal period promotes the development of immune tolerance, whereas microbial dysbiosis is associated with an increased risk of allergic diseases, autoimmune disorders, and infections (75). Ngo et al. demonstrates that the *segmented filamentous bacteria* reprogram AMs by altering their metabolic pathways, gene expression profiles, and functional characteristics. This reprogramming enables AMs to exert both antiviral and anti-inflammatory effects during respiratory viral infections, including IAV, respiratory syncytial virus (RSV), and SARS-CoV-2 (15). Similarly, Burrows et al. discovered that the gut commensal protozoan *Tritrichomonas musculis* establishes a tripartite immune network involving gut-derived inflammatory group 2 innate lymphoid cells, lung-resident T cells, and B cells (76). This network is formed through the remote reprogramming of the lung immune landscape, thereby influencing the outcomes of respiratory tract diseases. Furthermore, it has been confirmed that the BCG vaccine can induce long-lasting functional reprogramming of mature neutrophils. This reprogramming enhances their antimicrobial functions and increases their capacity to combat heterologous infections, such as those caused by the fungal pathogen *Candida albicans* (77).

## 5 Future perspectives of vaccine design for respiratory pathogens

### 5.1 The mucosal immune response serves as the first barrier of defense

It is widely recognized that numerous respiratory viruses can infect and replicate within both the upper and lower respiratory tracts, leading to damage at barrier sites and initiating inflammation-related tissue injury. Most of the currently approved vaccines for respiratory viruses, such as those for influenza and SARS-CoV-2, are administered at sites distant from the respiratory mucosa, such as intramuscularly or subcutaneously, to elicit systemic innate and adaptive immune responses. However, this approach may not provide sufficient protection at mucosal sites. Presently, there exists an immunological disconnect between the sites of vaccine administration (e.g., intramuscular or subcutaneous) and the primary routes of pathogen exposure (e.g., respiratory mucosa for airborne pathogens). Consequently, to effectively prevent infection and spread from becoming established in the first place, it is essential to develop vaccines capable of inducing protective mucosal immune responses in the respiratory tract (78, 79).

A previous study demonstrated that IgA plays a role in reducing viral load in the nasal passages and provides protection against influenza, despite low serum antibody titers in the volunteers. This finding underscores the significance of mucosal immune responses (80, 81). FluMist, a live-attenuated intranasal influenza vaccine, has been approved by the United States Food and Drug Administration (FDA) for self-administration in immunocompetent individuals aged 2 to 49 years (82). Studies have demonstrated that the FluMist vaccine effectively reduces influenza-related morbidity among children in daycare who are in contact with household members (83). Compared to the intramuscular inactivated vaccine, the FluMist vaccine has shown significantly greater efficacy in reducing laboratory-confirmed influenza in children (84). Moreover, the intranasal administration of a single-replication influenza vaccine can elicit mucosal secretory IgA (sIgA) and cell-mediated immune responses, which are correlates of protection in older adults (NCT05163847) (85). To investigate the relationship between protective efficacy and nasal mucosal antibody responses induced by the FluMist vaccine, Thwaites et al. conducted a clinical trial (NCT04110366). The study found that early mucosal immune responses were correlated with the extent of viral replication in the airway and were likely associated with protection (86).

During the initial development of COVID-19 vaccines, research predominantly focused on serum antibodies and cell-mediated immunity, often overlooking the crucial role of mucosal immunity. This oversight may account for the increased susceptibility to breakthrough infections and the shortened duration of protective immunity (87–89). In health care workers vaccinated with the wild-type SARS-CoV-2 spike, secretory circulating IgA antibodies were instrumental in preventing omicron infection (90). Furthermore, early mucosal IgA responses in respiratory tract tissues are vital for the initial control of SARS-CoV-2 infection, thereby preventing infection and subsequent transmission (91, 92).

Enhancing robust mucosal immune responses through use of safe and effective mucosal adjuvants is crucial for the advancement of mucosal vaccines. Presently, various attenuated or heat-killed bacteria are utilized as mucosal adjuvants, including *cholera toxin (CT)* (93) and *Escherichia coli heat-labile toxin* (94). An inhalable SARS-CoV-2 nanoparticle vaccine incorporating proteinaceous *CT* B subunits has demonstrated the ability to induce strong mucosal IgA immune response, underscoring the potential efficacy of mucosal adjuvants (95). Recent progress in genetic engineering has highlighted recombinant *lactic acid bacteria* and other commensal microbes as promising candidates for innovative mucosal vaccine delivery systems (96). By displaying predicted antigenic epitopes from the RBD of SARS-CoV-2 on platforms such as *Lactobacillus or Mycobacterium*, these mucosal vaccines effectively elicited strong IgA responses, optimizing for the addition of other adjuvants (97, 98). However, several critical issues must be considered when designing microbial mucosal expression vectors, including successful cloning within mucosal niches, the expression of heterologous proteins on the microbial surface, and maintaining genetic stability following genetic manipulation (99).

### 5.2 The influence of airway microbiota on respiratory health

As previously discussed, the gut microbiota plays a pivotal role in modulating adaptive immune responses to respiratory pathogens vaccines. Similarly, the airway microbiota, including nasal and oral bacteria, has been shown to confer innate antiviral resistance against respiratory pathogen infections. The respiratory tract and mucosal barrier sites host a diverse commensal microbiome that performs a crucial gatekeeping function in maintaining respiratory health through both cell-associated and secreted compounds (100, 101). Primarily, the mucus layer serves as a physical barrier, effectively preventing direct contact between pathogens and epithelial cells, thereby limiting pathogen colonization and invasion (102). Furthermore, airway epithelial cells secrete various antimicrobial proteins and peptides, such as lysozyme, which not only directly eliminate pathogens but also enhance host defense by modulating local immune responses (103). Another significant immunomodulatory molecule, secretory sIgA plays a crucial role in host defense by coating pathogens, thereby inhibiting their adherence to epithelial cells and facilitating their clearance, ultimately protecting the host from infection (100).

In comparison to the gastrointestinal tract, the respiratory tract harbors a lower microbial load and diversity (19). Nevertheless, the respiratory tract demonstrates microbial niche specialization, with the nasopharynx exhibiting the highest concentration of microorganisms. Consequently, certain microbiota colonizing the upper respiratory tract, such as *Streptococcus salivarius*, may have applications in the design of respiratory virus vaccines (99). Separated from human saliva and the dorsal surface of the tongue, *Streptococcus salivarius* is a predominant human commensal bacterium that initially colonizes the mucosa and predominates in the human oral cavity and upper airways (104). Through genetic engineering, this commensal bacterium has been successfully modified to target and elicit mucosal IgA in the respiratory tract by expressing specific antigens. A recent study explored the relationship between gut microbiota and the long-term immunogenicity of immunocompetent adults vaccinated with two doses of the SARS-CoV-2 BNT162b2 mRNA vaccine (105). The findings indicated that *Streptococcus salivarius* could serve as a predictor of long-term vaccine immunogenicity through its metabolic pathway markers, underscoring its potential as a mucosal vector vaccine that could enhance the immune system. Interestingly, some commensal oral bacterial strains such as *Streptococcus salivarius*, have demonstrated efficacy as adjuvants in enhancing mucosal immune responses. When supplemented with cultured oral bacteria *Streptococcus salivarius*, intranasal vaccination with IV and SARS-CoV-2 vaccines elicited significant antigen-specific nasal IgA and serum IgG responses in a MyD88-dependent manner (106). However, achieving an optimal balance between respiratory resistance and tolerance within the mucosal immune environment poses a challenge for incorporating airway microbiota into respiratory virus vaccine design. For instance, individuals with allergic rhinitis (AR) exhibit an increased abundance of *Streptococcus salivarius* compared to healthy individuals (107).

Moreover, commensal bacteria and pathogens often share conserved antigenic epitopes, such as the linear epitope homology between *Streptococcus pneumoniae* PspA and the IV HA protein, which facilitates cross-reactive immune responses (108). Future vaccine strategies could involve engineering commensal bacteria to present pathogen-conserved antigens, thereby creating “one vaccine for multiple diseases” while utilizing the natural adjuvant properties of microbes to minimize toxicity.

### 5.3 Mucosal immune synergy driven by the crosstalk gut-lung axis

The bidirectional regulation of the gut-lung axis provides a theoretical basis for the development of next-generation mucosal vaccines. The gut microbiota activates the gut-associated lymphoid tissue (GALT), leading to the migration of CD103^+^ dendritic cells (DCs) to lung-draining lymph nodes and promoting the differentiation of follicular helper T cells (Tfh), which in turn drive B cell class-switching to sIgA. The live attenuated BCG vaccine has been shown to generate memory AMs and induce trained immunity in the lungs through cross-organ immune regulation via the gut-lung axis, thereby offering protection during the early stages of *Mtb* infection (38). This study suggests that the replicative capacity of live attenuated BCG is crucial for eliciting systemic immune responses at distant sites. Live attenuated vaccines may more effectively induce systemic immune activation, coordinating complex immune responses across tissues through microbial metabolite and cytokine networks. Future respiratory vaccine design could prioritize live vectors, such as modified vaccinia Ankara (MVA), to maximize the synergistic effects of the gut-lung axis (109).

Intranasal SARS-CoV-2 vaccination emulates natural infection by directly engaging mucosal M cells and epithelial cells, thereby inducing tissue-resident memory T cells (TRMs) within the airway epithelia, which facilitates rapid secondary immune responses (110, 111). In contrast, conventional intramuscular vaccines frequently fail to elicit mucosal immunity, resulting in breakthrough infections. To address this, innovative delivery systems must incorporate microbiome characteristics. Technologically, the integration of microbiome-derived molecules with novel delivery systems presents substantial potential. Adhesins secreted by the respiratory microbiota, such as the *Streptococcus* surface protein PilA, facilitate cross-mucosal antigen transport (112). Therefore, it is plausible to design a nasal vaccine that enhances pulmonary IgA titers by conjugating the target antigen with PilA. Furthermore, certain studies indicate that the PsaA protein of *Streptococcus pneumoniae* interacts with Annexin A2 on human airway epithelial cells, promoting bacterial colonization (113). These investigations elucidate the complex roles of adhesins in host-pathogen interactions, offering novel insights for the development of vaccines and therapeutic strategies against respiratory viral infections. For example, future vaccines may be combined with the use of gut commensal microbiota such as *segmented filamentous bacteria*, *Tritrichomonas musculis*, which is beneficial to enhance the mucosal immune synergy driven by the gut-lung axis crosstalk.

## 6 Conclusion

The gut and respiratory microbiomes are transforming the design of respiratory vaccines by influencing metabolic regulation, mucosal synergy, and personalization. Strategies such as SCFA-enhanced DCs maturation, engineered symbiotic delivery systems, and microbiome-tailored precision approaches are being developed to enhance vaccine efficacy and durability, while also addressing viral diversity and the needs of vulnerable populations. With the integration of single-cell multi-omics, systems vaccinology, and artificial intelligence, next-generation vaccines are expected to shift from “broad-spectrum coverage” to “precision targeting.” (114, 115)
